# Tamoxifen and the Singing Voice

**DOI:** 10.1371/journal.pmed.0020310

**Published:** 2005-09-27

**Authors:** Andrew Herxheimer

**Affiliations:** **1**United Kingdom Cochrane CentreOxfordUnited Kingdom

My remark, in my recent Essay in *PLoS Medicine* [1], that deepening of the voice occurs with long-term use of tamoxifen for breast cancer needs qualification.

Several colleagues have rightly pointed out that the evidence for the effect is less clear than I implied: it comes from women who have experienced it [2], but there have been no controlled studies. A change in voice was looked for and not found among effects spontaneously reported in large trials of tamoxifen, but this was not specifically asked about and might well have been missed. It is also recognised that the voice sometimes becomes deeper during or after menopause, in the absence of tamoxifen.

To convey the uncertainty of the facts, I wish to amend my statement as follows: “The irreversible deepening of the voice that has been reported to occur with long-term use of tamoxifen for breast cancer is an example of a side effect that prescribers, manufacturers, and drug regulators seem to have considered trivial and have not investigated.”
